# Predicting Physical Appearance from Low Template: State of the Art and Future Perspectives

**DOI:** 10.3390/genes17010059

**Published:** 2026-01-05

**Authors:** Francesco Sessa, Emina Dervišević, Massimiliano Esposito, Martina Francaviglia, Mario Chisari, Cristoforo Pomara, Monica Salerno

**Affiliations:** 1 Department of Psychology and Health Sciences, Faculty of Human Sciences, Education, and Sports, Pegaso University, 80143 Naples, Italy; 2 Department of Forensic Medicine, Faculty of Medicine, University of Sarajevo, 71000 Sarajevo, Bosnia and Herzegovina; emina.dervisevic@mf.unsa.ba; 3 Faculty of Medicine and Surgery, “Kore” University of Enna, 94100 Enna, Italy; massimiliano.esposito@unikore.it (M.E.); mario.chisari@unikore.it (M.C.); 4 Department of Medical, Surgical and Advanced Technologies “G.F. Ingrassia”, University of Catania, 95121 Catania, Italy; martinafrancaviglia95@gmail.com (M.F.); cristoforo.pomara@unict.it (C.P.)

**Keywords:** forensic DNA phenotyping (FDP), low template DNA (LT-DNA), machine learning (ML), externally visible characteristics (EVC), SNP-based prediction, forensic genomics

## Abstract

**Background/Objectives**: Forensic DNA phenotyping (FDP) enables the prediction of externally visible characteristics (EVCs) such as eye, hair, and skin color, ancestry, and age from biological traces. However, low template DNA (LT-DNA), often derived from degraded or trace samples, poses significant challenges due to allelic dropout, contamination, and incomplete profiles. This review evaluates recent advances in FDP from LT-DNA, focusing on the integration of machine learning (ML) models to improve predictive accuracy and operational readiness, while addressing ethical and population-related considerations. **Methods**: A comprehensive literature review was conducted on FDP and ML applications in forensic genomics. Key areas examined include SNP-based trait modeling, genotype imputation, epigenetic age estimation, and probabilistic inference. Comparative performance of ML algorithms (Random Forests, Support Vector Machines, Gradient Boosting, and deep learning) was assessed using datasets such as the 1000 Genomes Project, UK Biobank, and forensic casework samples. Ethical frameworks and validation standards were also analyzed. **Results**: ML approaches significantly enhance phenotype prediction from LT-DNA, achieving AUC > 0.9 for eye color and improving SNP recovery by up to 15% through imputation. Tools like HIrisPlex-S and VISAGE panels remain robust for eye and hair color, with moderate accuracy for skin tone and emerging capabilities for age and facial morphology. Limitations persist in admixed populations and traits with polygenic complexity. Interpretability and bias mitigation remain critical for forensic admissibility. **Conclusions**: L integration strengthens FDP from LT-DNA, offering valuable investigative leads in challenging scenarios. Future directions include multi-omics integration, portable sequencing platforms, inclusive reference datasets, and explainable AI to ensure accuracy, transparency, and ethical compliance in forensic applications.

## 1. Introduction

The forensic application of genetic data has evolved from a tool of straightforward identity verification to a complex system capable of predicting human phenotypes from biological traces, a field known as Forensic DNA Phenotyping (FDP) [1,2]. This paradigm shift is driven by technological advances in genomics, particularly genome-wide association studies (GWAS), next-generation sequencing (NGS), and increasingly sophisticated computational modeling [3]. FDP allows investigators to derive investigative leads from crime scene DNA by predicting externally visible characteristics (EVCs) such as eye, hair, and skin color, biogeographic ancestry, and chronological age, even in the absence of a known reference profile [4].

The idea of forensic DNA intelligence is to extract any information from DNA that can help guide criminal investigations, especially when no match exists in short tandem repeat (STR) databases [5]. Of practical importance are clues to the physical phenotype, due to the high heritability of human appearance traits [6]. While early forensic genetics relied on STR profiling for identity matching, STR markers are neutral and do not provide phenotypic information [7]. FDP has thus emerged as an essential tool in cases involving unidentified perpetrators, cold cases, mass disasters, or heavily degraded biological remains [8].

The notion that physical features can be “read” from DNA began to gain scientific credibility in the early 2000s with the publication of large-scale GWAS that identified single nucleotide polymorphisms (SNPs) associated with human pigmentation and morphology. Early SNP-based systems such as IrisPlex and HIrisPlex-S demonstrated the feasibility of predicting eye and hair color with moderate to high accuracy. As understanding of complex trait genetics expanded, FDP broadened to include traits like freckles, hair structure, male pattern baldness, and tall stature, as well as subcontinental ancestry and age from multiple tissues [1].

**Figure 1:**
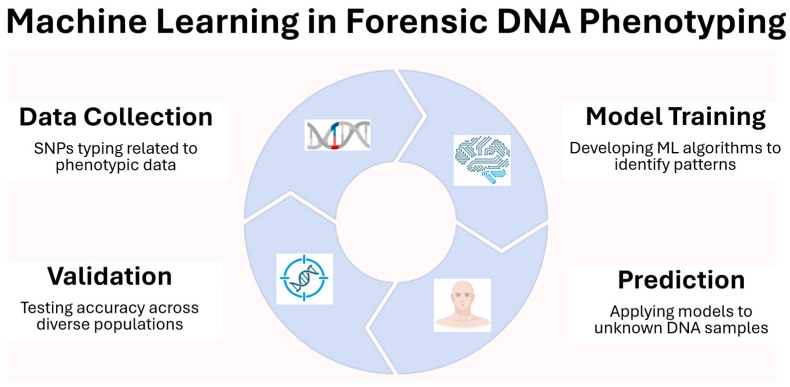
The integration of ML with FDP has significantly enhanced the ability to extract meaningful information from LT-DNA, though the accuracy of predictions remains fundamentally tied to data quality, genetic coverage, and technological sensitivity (created with BioRender, https://www.biorender.com).

A major challenge in FDP is the prediction of complex traits, which are polygenic and often influenced by environmental and developmental factors. Traits like facial morphology or skin tone involve many small-effect variants, requiring big data approaches and machine learning (ML) models trained on large, diverse reference datasets (Figure 1). For simpler traits with high heritability, targeted analysis of a limited number of markers using massively parallel sequencing (MPS) has proven effective. However, achieving universal genomic predictors for all traits will require the construction of much larger databases derived from whole-genome sequencing (WGS), including rare variants that may influence phenotypes. Furthermore, for accurate appearance prediction, age estimation must be considered; this can be derived from epigenetic modifications, particularly DNA methylation markers, which reflect biological age [3].

Technological limitations persist, especially in cases involving LT-DNA or degraded skeletal remains, which are common in real-world forensics. LT-DNA samples, often collected from trace biological material such as epithelial cells, touched objects, or aged remains, are susceptible to allelic dropout, amplification errors, and partial genotyping. Despite these challenges, tools like the Ion AmpliSeq™ HIrisPlex-S panel, validated using Ion Torrent MPS technology, have shown remarkable resilience. In a study analyzing 63 bone samples aged up to 80 years, full pigmentation profiles could be recovered from over 55% of cases, with successful eye and hair color predictions made from partial profiles in many others. Nonetheless, certain SNPs, especially those relevant to skin color, showed consistent underperformance, highlighting the need for primer redesign in future iterations of the panel [9].

Importantly, the correctness and utility of forensic phenotyping are fundamentally tied to data quality, genetic coverage, and technological sensitivity. As shown by the example of paleoanthropology, where DNA is extracted from highly degraded remains, there is potential for adapting high-throughput, low-input genomic methods to forensic contexts. The slow pace of their forensic adoption, however, is due in part to technical, regulatory, and funding constraints [10,11,12,13,14].

This review explores the scientific and technological advances that are reshaping the field of FDP, with particular emphasis on the application of ML for predicting physical appearance from LT-DNA. It begins by defining LT-DNA and outlining the unique challenges it presents in forensic investigations. The development of FDP is then examined, from its early use in identity testing to its current application in predicting (EVCs), biogeographic ancestry, and chronological age. Particular attention is given to the integration of ML approaches, which have become essential for modeling complex traits from partial or degraded genetic data. The review also addresses the practical and technical limitations associated with compromised samples and highlights how recent technological and computational advancements aim to mitigate these issues. Finally, it discusses future perspectives in the field, including the integration of multi-omics data, the expansion of reference databases to enhance predictive accuracy across diverse populations, and the ethical considerations surrounding the forensic use of predictive genetic information.

## 2. Low Template DNA: Challenges and Opportunities

### 2.1. Definition and Characteristics

LT-DNA represents a critical category of forensic genetic evidence characterized by a minute quantity of recoverable DNA, typically considered to be below 100 picograms (pg), or fewer than approximately 15–20 human diploid cells [15]. Such low-level samples are frequently encountered in crime scene investigations involving biological trace, including touch DNA, aged stains, shed hairs, or severely degraded tissue [16]. The forensic utility of LT-DNA lies in its potential to provide investigative leads in cases where standard DNA profiling techniques may fail due to insufficient template quantity, stochastic effects, or contamination risks [17].

A defining feature of LT-DNA analysis is its susceptibility to stochastic variation during PCR amplification, leading to challenges such as allelic dropout (failure to detect one allele of a heterozygous locus), allelic drop-in (spurious alleles introduced during amplification), and peak imbalance in electropherograms [18]. These artifacts compromise interpretation reliability, necessitating both technical rigor and statistical caution in forensic applications. To address these limitations, laboratories have developed specialized protocols and technologies aimed at maximizing the information recovered from low-level DNA while minimizing noise and erroneous profiles [19].

One of the central technical questions in processing LT-DNA is whether to concentrate the available extract into a single amplification or split it across replicates. A decision-theoretical study investigating this dilemma demonstrated that performing two replicates yields a higher expected net gain (ENG) than a single amplification, provided that the DNA quantity is sufficient to produce peak heights as low as 43 relative fluorescence units (RFU) on average. This finding underscores the benefit of replicate analyses in enhancing the reliability and information content of LT-DNA profiles, particularly when operating near the analytical sensitivity threshold of current instrumentation [20].

**Figure 2:**
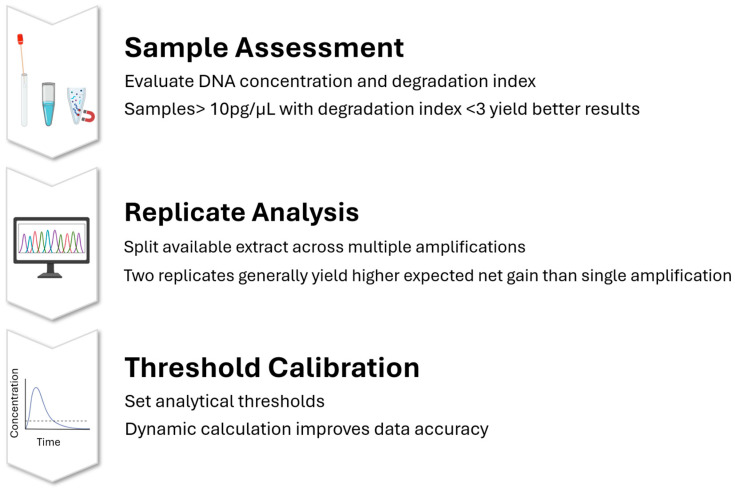
Optimized workflow for LT-DNA analysis in forensic laboratories. Key steps include: (1) sample assessment for DNA concentration and degradation index; (2) replicate analysis to improve reliability and ENG; and (3) dynamic threshold calibration based on baseline signal distributions. (Created with BioRender: www.biorender.com).

Analytical threshold (AT) determination is another key factor in managing LT-DNA profiles. Given the low signal intensity and high background noise often observed in such samples, accurate AT setting becomes crucial for distinguishing true alleles from spurious peaks. Research has shown that baseline signal patterns are influenced by reagent kits, laboratory conditions, instrument calibration, and the number of amplification cycles. In response, some laboratories now calculate the AT dynamically using baseline signal distributions from negative control profiles. One study proposed a comparative framework for evaluating AT methods and introduced a real-time analysis tool that allows forensic analysts to tailor ATs to specific experimental conditions, improving data accuracy while preserving true allele signals in most LT-DNA scenarios, though challenges persist in extremely low-template and high-cycle cases [21,22] (Figure 2).

Beyond analytical thresholds, optimizing amplification strategies is essential when dealing with limited or degraded DNA. A study involving 155 forensic case samples with DNA concentrations between 5 and 14.3 pg/μL revealed that samples exceeding 10 pg/μL and with a degradation index below 3 (a ratio indicating the relative amplification success of short versus long DNA fragments, used to assess sample degradation) were significantly more likely to yield informative profiles. Applying empirically derived thresholds for DNA quantity and quality, researchers found that forensic laboratories could reduce analytical workload by 32% while retaining 83% of usable profiles, offering a pragmatic approach to resource allocation in high-throughput forensic contexts [23].

Recent developments in post-PCR processing have also demonstrated promises for improving allele recovery from low template and trace DNA samples. A study evaluating the Amplicon RX post-PCR clean-up method found significant improvements in allele recovery and signal intensity when compared to standard 29- and 30-cycle protocols using the GlobalFiler amplification kit. Particularly at DNA concentrations as low as 0.001 ng/μL (i.e., 1 pg/μL), the Amplicon RX method yielded superior results, reinforcing its value in analyzing extremely low-template and degraded samples. While all methods struggled at the lowest tested concentration (0.0001 ng/μL), the clean-up procedure still outperformed traditional protocols, emphasizing the need for continued innovation in post-amplification refinement techniques [24].

Despite these methodological advances, significant limitations remain in working with LT-DNA. The inherent stochasticity of low-copy amplification, variability in laboratory-specific workflows, and the impact of environmental factors such as temperature and humidity all contribute to result uncertainty [25,26]. Additionally, inter-laboratory standardization for defining what constitutes “low template” remains inconsistent, although the commonly cited threshold of 100 pg has become widely accepted as a working guideline [27]. Importantly, successful profiling from LT-DNA is not solely a function of DNA quantity, but rather a balance of factors including degradation, inhibition, locus-specific amplification efficiency, and the effectiveness of downstream analysis tools [28].

In conclusion, LT-DNA represents both a challenge and an opportunity in forensic genetics. Its successful analysis requires careful consideration of DNA quantity, quality, amplification strategies, threshold calibration, and post-PCR treatment. The body of evidence now supports the use of multi-replicate strategies, adaptive thresholding, and optimized amplification-clean-up protocols as key methods to enhance the reliability of LT-DNA profiling. As technological advancements in sequencing sensitivity and forensic assay design continue to evolve, it is anticipated that the definition and analytical handling of LT-DNA will become increasingly standardized and robust, improving the evidentiary value of this critical class of forensic samples.

### 2.2. Sources of LT-DNA

LT-DNA is frequently obtained from minute or degraded samples encountered in forensic casework, including touch DNA, shed or rootless hair shafts, trace biological fluids, and skeletal remains such as aged bone and teeth. While these sources can be pivotal in linking a suspect to a crime scene or identifying unknown remains, they each present distinct challenges related to DNA quantity, degradation, contamination risk, and extraction complexity [29,30].

Touch DNA, or transfer DNA, is one of the most common sources of LT-DNA in forensic casework. It consists of DNA deposited via skin contact on surfaces, items, or human skin. The DNA is primarily derived from shed epithelial cells (corneocytes), which are often anucleated or contain degraded nuclear material. Studies have shown that to recover a full STR profile using direct PCR, at least 40 buccal cells or 4000 corneocytes are needed from touch samples collected via swab or tape lift. When extracted using conventional workflows, 8000 or more corneocytes may be required to obtain comparable results [31]. Collection methods significantly impact DNA recovery, though no single swabbing technique has proven superior. The widely used double-swab method showed modest improvement in DNA yield (average 13.7% more DNA) but not necessarily in STR recovery [32]. Further complicating touch DNA analysis is the indirect transfer of human DNA into the environment, onto dust, furniture, or air particles, raising the possibility of retrieving DNA from individuals who were merely present in a room, not necessarily involved in a criminal act [33].

Hair shafts, especially shed or rootless hairs, are another key yet problematic source of LT-DNA. Hair shafts contain highly degraded and fragmented nuclear DNA, often in insufficient quantities for STR profiling, particularly when subjected to environmental stress such as UV exposure or chemical treatment [34]. While mitochondrial DNA (mtDNA) can be recovered more consistently from hair shafts, hair nuDNA typing has been improved by massively parallel sequencing (MPS) platforms, which enable simultaneous analysis of mtDNA, STRs, SNPs, and Amelogenin markers [35]. DNA extraction methods, such as the Investigator and MinElute protocols, significantly affect outcomes; the Investigator method has shown superior results in terms of depth of coverage, SNP genotyping success, and lower dropout rates [35]. Additionally, telogen hair roots, those not in active growth phase, have historically yielded poor DNA results. However, using Hematoxylin staining to quantify nuclear content before selecting roots for testing has been shown to double the success rate of DNA profiling, from 32% to 69% [36].

Trace biological fluids such as saliva, sweat, and dried blood smears are also common sources of LT-DNA. These samples often contain small quantities of nucleated cells, and the DNA yield varies widely. Fluids such as urine and sweat yield the least DNA due to their low cellular content, whereas blood and semen typically contain higher concentrations of nucleated cells and are more amenable to traditional STR analysis [37]. Identification and proper collection of such fluids, especially when partially degraded or exposed, are essential to ensure successful analysis. Innovative strategies using fluorescent nucleic acid dyes have enhanced the visualization and targeted collection of cellular material, improving downstream processing success rates [38].

Skeletal remains, including bones and teeth, are routinely encountered in forensic investigations involving long postmortem intervals or extreme environmental exposure. These matrices are inherently challenging due to the degradation of DNA over time by temperature, humidity, microbial activity, and pH. Among skeletal elements, the petrous part of the temporal bone has demonstrated the highest DNA yield, up to 30 times that of other bones, due to its dense structure and protective anatomy. Several studies have confirmed that petrous bones outperform femurs, metacarpals, and even teeth in terms of STR profile completeness and reduced dropout rates [39,40]. Similarly, teeth remain a viable DNA source even when exposed to harsh environmental or chemical conditions, such as prolonged submersion in acids, due to their resilient enamel structure. Full or partial DNA profiles can still be recovered from teeth exposed to sulfuric, hydrochloric, or nitric acids for up to 120 h [41].

Aged or degraded skeletal samples require optimized pre-analytical strategies, including the use of decalcification agents, silica-based extraction protocols, and automated systems such as the Maxwell FSC DNA IQ platform. These methods improve yield and reduce handling time, though the success of DNA recovery still largely depends on selecting the most suitable skeletal elements and applying strict contamination controls [42].

In conclusion, LT-DNA is obtained from a wide range of forensic materials, each presenting unique challenges that must be addressed through tailored extraction, quantification, and amplification protocols. The advent of MPS technologies, nuclear content screening, and enhanced collection methods has significantly expanded the ability to recover probative genetic information from even the most limited and compromised DNA sources. Continued innovation in these areas, combined with better triage methods and decision thresholds, will be essential for maximizing the utility of LT-DNA in forensic casework.

### 2.3. Current Forensic Approaches for LT-DNA Analysis

LT-DNA analysis involves working with very small amounts of DNA, and presents a series of challenges related to sensitivity, reproducibility, and interpretation. Despite these limitations, significant advances in molecular techniques and analytical strategies have allowed forensic scientists to maximize the evidentiary value of minimal and degraded DNA samples, which are increasingly encountered in real-world forensic contexts.

One of the central technical approaches in LT-DNA analysis is the increase in PCR cycle numbers during STR amplification to enhance sensitivity. By extending the amplification process beyond the standard 28–30 cycles to 34 or more, low levels of DNA can be more effectively amplified, increasing the likelihood of detecting alleles that would otherwise remain below analytical thresholds. However, this approach is not without drawbacks. Stochastic effects, including allelic and locus drop-out, allelic drop-in, and peak imbalance, become more pronounced as the DNA quantity decreases and amplification cycles increase. These artifacts can compromise the reliability of the resulting profiles, especially in mixed samples or degraded material. Nonetheless, validation studies have demonstrated that replicate testing, even at very low template levels (e.g., 10–100 pg), can produce reliable single-source profiles when a consensus approach is used. Such strategies have helped forensic laboratories implement protocols that balance sensitivity with interpretive robustness, even in high-stakes criminal investigations [43].

In addition to replicating testing, alternative strategies for improving the quality of LT-DNA results have gained traction. Among these is single-cell DNA (scDNA) analysis, which offers a solution to the limitations of traditional “bulk” extraction methods used for mixed samples. In conventional workflows, crime scene stains containing multiple contributors are extracted en masse, producing a homogenized mixture that complicates deconvolution. In contrast, scDNA approaches involve subsampling individual cells from the mixture before DNA extraction, thereby enabling the generation of highly probative, single-source DNA profiles. This technique is particularly advantageous in resolving complex mixtures, such as those involving relatives with similar genetic profiles, or when detecting low-level contributors who may be masked in standard bulk analysis. Although scDNA techniques are still in development and face technical hurdles related to sensitivity, contamination risk, and cost, they represent a promising direction for the future of LT-DNA analysis and forensic genomics as a whole [15].

Beyond individual technical innovations, forensic laboratories are also integrating probabilistic genotyping software (PGS) to enhance interpretation of low-template or complex DNA profiles. These software platforms employ statistical modeling to assign likelihood ratios to competing hypotheses about DNA contributors, incorporating drop-out probabilities, stutter modeling (stutter refers to minor PCR artifacts where a small peak appears one repeat unit shorter than the true allele, caused by slippage during amplification), and peak height variability. In LT-DNA contexts, where traditional binary interpretation methods often fall short, PGS allows analysts to extract meaningful conclusions from incomplete or uncertain data. Probabilistic genotyping is now considered the best practice in the analysis of complex mixtures and degraded samples and is increasingly used in courtrooms worldwide [44].

Another major advancement supporting LT-DNA analysis is the implementation of NGS, which enables massively parallel processing of STRs, SNPs, and other markers from minimal input DNA. NGS offers several advantages over capillary electrophoresis (CE)-based STR analysis, including the ability to detect additional sequence variation within STR alleles, improved resolution of mixture components, and simultaneous analysis of mitochondrial and nuclear DNA. In LT-DNA contexts, NGS improves both the quantity and quality of data recovered from challenging samples, such as aged bones, hair shafts, or trace skin cells. However, NGS is not yet universally adopted in forensic laboratories due to its cost, complexity, and validation requirements. Continued technological refinement and standardization will be essential for widespread implementation in routine casework.

Despite these technical advances, LT-DNA analysis is not without controversy. Ethical and legal debates persist regarding the admissibility and interpretation of low-level DNA evidence. Concerns about contamination, false inclusions, and the risk of overstating the probative value of partial profiles have led courts and scholars to scrutinize the scientific reliability and legal fairness of LT-DNA testimony. Furthermore, as forensic DNA analysis becomes more powerful and intrusive, especially with the rise in familial searching, phenotyping, and investigative genetic genealogy, important questions arise about privacy, consent, and the potential infringement of civil liberties. Drawing on historical parallels from the early days of DNA profiling, some commentators argue that current advances must be met with a renewed commitment to protecting individual rights and ensuring transparent, accountable use of forensic science [43].

Looking ahead, the future of LT-DNA analysis lies in the integration of multidisciplinary approaches. As highlighted by the INTERPOL Forensic Science Managers Symposium, the field is advancing through the convergence of technologies such as rapid DNA testing, lineage marker analysis, microhaplotypes, proteomics, and epigenetic clocks for age prediction. These methods are being combined with enhanced extraction protocols, automation, and increasingly sophisticated bioinformatic tools to overcome the inherent limitations of low-template samples. Quality assurance remains a top priority, with over 70 new international guidance documents published in recent years to standardize procedures, enhance reliability, and promote cross-laboratory reproducibility [44].

In conclusion, current forensic approaches in LT-DNA analysis represent a dynamic and rapidly evolving area of forensic science. Through the combined use of increased PCR cycles, replicate consensus profiling, probabilistic modeling, single-cell analysis, and NGS technologies, laboratories are increasingly capable of obtaining meaningful results from the smallest and most degraded samples. At the same time, the legal and ethical frameworks surrounding these technologies must evolve in parallel to ensure that forensic innovations serve justice while respecting fundamental rights.

## 3. From Genotype to Phenotype: The Rise in FDP

The emergence of FDP marks a paradigm shift in modern forensic science. Where traditional DNA profiling has long been limited to identifying individuals through STR markers, FDP opens the possibility of predicting EVCs directly from genetic material, particularly in human remains [45]. By bridging the gap between genotype and phenotype, FDP enables investigators to infer aspects of an unknown person’s physical appearance, ancestry, and even age from DNA found at a crime scene [46]. This approach offers valuable investigative leads, especially in cases where conventional methods reach a dead end [1].

FDP currently focuses on a defined but gradually expanding set of traits, each with its own level of scientific maturity and predictive accuracy. Eye color, for instance, is among the most reliable phenotypic traits to be predicted from DNA, thanks to well-characterized variants in genes such as *HERC2* and *OCA2* [47,48]. In contrast, traits like skin pigmentation and hair color are more genetically complex, influenced by multiple genes and moderated by environmental factors. While red and black hair can be predicted with reasonable confidence, intermediate hair shades and skin tones remain more difficult to assess, particularly in admixed or non-European populations [49,50,51].

Secondary traits such as freckling and sun sensitivity can further refine a DNA-based appearance profile, though they are typically used in conjunction with primary pigmentation traits [52]. A more ambitious goal is the prediction of facial morphology, which remains one of the most challenging areas due to its highly polygenic nature and sensitivity to non-genetic influences like age, nutrition, and development. Although some genetic variants (e.g., in *PAX3* or *EDAR*) have been associated with facial shape, practical applications in forensic casework are still in the early stages [53].

In parallel, advancements in epigenetics have introduced the possibility of estimating chronological age from DNA, using methylation markers at specific CpG sites. Though age is not a fixed phenotype in the genetic sense, these DNA methylation-based “epigenetic clocks” offer promising tools for narrowing suspect or victim pools, particularly when traditional identifiers are absent [54]. Moreover, biogeographical ancestry inference provides essential context for phenotype prediction, as genetic variant frequencies differ across populations [55].

Tools like IrisPlex, HIrisPlex, and HIrisPlex-S panels, as well as the VISible Attributes through GEnomics (VISAGE) Enhanced Tool, have made FDP more accessible in forensic settings, allowing for targeted genotyping of key SNPs related to eye, hair, and skin color. Genotyping 24 genetic markers (SNPs and indels) enables rapid and reliable prediction of eye and hair color using the HIrisPlex system; the inclusion of 17 additional markers extends this capability to skin color prediction through the HIrisPlex-S system. While these panels show strong performance under ideal laboratory conditions, their predictive accuracy often diminishes in real-world scenarios involving degraded, mixed, or low-template DNA. As a result, integrating FDP into investigations requires not only scientific rigor but also careful consideration of its limitations and the probabilistic nature of the predictions it offers [56,57,58,59]. It is important to note that current forensic prediction panels (IrisPlex, HIrisPlex-S, VISAGE) primarily employ multinomial logistic regression for trait inference. These models are interpretable and validated for forensic use but differ fundamentally from ML approaches discussed later, which aim to capture complex genotype–phenotype relationships using non-linear, high-dimensional methods.

### 3.1. Forensic Eye Color Prediction from DNA: Insights from Genetic Markers

In forensic science, biological traces such as blood, saliva, semen, or epithelial cells recovered from crime scenes are primarily used for human identification through DNA profiling. However, recent advances have enabled the use of DNA to predict EVCs [60].

Eye color prediction is based on the analysis of specific SNPs associated with pigmentation genes [47]. These SNPs influence melanin synthesis and distribution in the iris, which, despite being covered by the transparent cornea, exhibits color variation due to both pigment concentration and structural light scattering in the stroma. The variation in iris color is primarily determined by the number and distribution of stromal melanocytes and the type of melanin, especially eumelanin, present [61].

Eye color is a polygenic trait, with multiple genes contributing to its expression [57]. Among these, the SNP rs12913832 in the *HERC2* gene has the most significant impact, particularly in European populations. Although located in an intronic region of *HERC2*, this SNP regulates the expression of the *OCA2* gene, which encodes a protein essential for melanin transport. Individuals with the AA genotype at rs12913832 typically have blue eyes, GG genotype correlates with brown eyes, and AG heterozygotes often exhibit intermediate shades such as green or hazel [62,63].

Other genes, such as *SLC24A4*, *SLC45A2*, *TYR*, *TYRP1*, *ASIP* or *IRF4*, also contribute to eye color variation, especially for intermediate phenotypes. However, their predictive power is generally lower and may vary across populations. Polymorphisms in these genes, while informative, are often population-specific and less conserved than the *HERC2-OCA2* regulatory axis [64,65,66].

To operate these genetic insights, the IrisPlex system was developed. This forensic tool uses a panel of six SNPs (Table 1) to predict eye color using a multinomial logistic regression model. The system outputs probabilities for three eye color categories: blue, brown, and intermediate. A prediction is considered reliable when the highest probability exceeds a threshold of 0.7. For example, a prediction of 0.89 for blue, 0.08 for brown, and 0.03 for intermediate would be interpreted as blue eyes [58].

Validation studies have demonstrated that IrisPlex achieves over 90% accuracy for blue and brown eyes in European populations. However, prediction accuracy for intermediate eye colors remains lower (around 73%), with sensitivity as low as 1.1%, reflecting the complex genetic architecture of these phenotypes [58].

As detailed in Section 2.3, LT-DNA introduces stochastic effects and allelic dropout that may compromise prediction accuracy. Future challenges in iris color prediction lie in integrating newly identified genetic markers to enhance predictive accuracy. A recent publication highlighted that whole-exome sequencing of 150 individuals has uncovered 27 previously unreported variants associated with eye color, offering promising new targets for prediction models. Notably, the SNP rs2253104 in the *ARFIP2* gene emerged as a key predictor, selected by multiple feature selection methods and contributing to the most accurate regression models. These findings suggest that expanding SNP panels with newly validated variants could significantly improve prediction outcomes, especially when working with degraded or limited DNA samples where maximizing the information from each locus is crucial. Validating and adapting these new markers for use in mini-amplicon assays could be a key step forward in applying iris prediction to challenging forensic samples [47].

Additionally, high-sensitivity genotyping platforms, including MassARRAY (MALDI-TOF mass spectrometry for multiplex SNP genotyping), NGS/MPS (parallel sequencing of multiple loci), and real-time PCR (using allele-specific probes or high-resolution melting analysis), have enhanced the precision and robustness of SNP detection [51,67]. Replicate testing and consensus genotyping further mitigate random amplification errors by allowing analysts to identify consistent results across multiple runs. Moreover, the integration of probabilistic models into prediction tools enables the communication of uncertainty levels, helping to avoid overinterpretation of marginal or ambiguous profiles. Enforcing strict quality control thresholds, such as minimum allele calling criteria and prediction probability cutoffs (e.g., >0.7), also ensures that only high-confidence phenotype predictions are reported. Nevertheless, certain limitations remain. Intermediate eye colors, for instance, continue to be difficult to predict accurately due to their polygenic inheritance patterns and relatively low heritability. Furthermore, variations in allele frequencies across populations can influence prediction outcomes, highlighting the necessity of validating predictive models in diverse genetic backgrounds to ensure their broader applicability and forensic reliability [68].

While eye color is generally considered a low-risk trait in terms of privacy, its use in FDP must adhere to ethical standards and legal frameworks. Misinterpretation of probabilistic predictions, especially in cases with low confidence or ambiguous results, can lead to investigational bias. Therefore, careful communication of FDP findings to law enforcement is crucial to avoid wrongful suspicion [11,69].

### 3.2. Hair Color Prediction from Biological Traces

When biological traces are collected, forensic experts apply extraction protocols designed to preserve DNA quality even from degraded or low template samples [70]. Following DNA isolation and quantification, molecular techniques are used to identify SNPs associated with hair pigmentation [71].

Specific DNA regions are involved in the production, distribution, and degradation of melanin, the pigment primarily responsible for hair, eye, and skin coloration [56]. Hair color is determined by the type and quantity of melanin in the hair shaft: eumelanin (black/brown pigment) and pheomelanin (red/yellow pigment). The balance between these two types of melanin, regulated by a network of genes and genetic variants, gives rise to the wide spectrum of human hair colors, from black and brown to blond and red [72].

Hair color is a complex trait shaped by the combined influence of many genes and their interactions, each contributing in different ways to the final pigmentation phenotype. Among these, *MC1R* (Melanocortin 1 Receptor) plays a particularly prominent role. Variants in this gene are closely linked to red hair, as *MC1R* controls the switch between the production of eumelanin (dark pigment) and pheomelanin (red/yellow pigment). When *MC1R* function is disrupted by specific genetic variants, the balance shifts toward pheomelanin, leading to the characteristic red or auburn hair tones [73,74,75].

Other genes also contribute significantly to hair color diversity. *HERC2* and *OCA2*, which are more widely recognized for their role in determining eye color, also influence melanin synthesis in the hair, particularly affecting the variations observed in blond and brown shades [48].

Genes such as *SLC24A4*, *SLC45A2*, and *TYR* further support the pigmentation process by regulating melanosome function and the activity of tyrosinase, an enzyme critical for melanin biosynthesis. Variants in these genes are commonly associated with lighter hair tones [76].

Lastly, *IRF4* has emerged as another important contributor. This gene influences pigmentation through its regulatory functions in melanocyte biology. Not only is it associated with lighter hair colors, but it is also implicated in age-related changes in pigmentation, such as the gradual transition to gray hair [77].

The HIrisPlex system, developed as an extension of the earlier IrisPlex model for eye color prediction, integrates both eye and hair color SNPs into a single assay. As summarized in Table 2, the system uses 24 SNPs across multiple pigmentation genes [56].

The model outputs probability for each color, allowing forensic experts to assess the likely hair color of the DNA donor. A prediction is usually accepted when one color category exceeds a predefined probability threshold (e.g., >70%). For example, a sample yielding 0.82 probability for brown, 0.10 for black, 0.06 for blond, and 0.02 for red would result in a brown hair color prediction [78].

The predictive accuracy of hair color varies by color category and population group. For example, red hair can be predicted with over 80–90% accuracy, primarily due to the strong effect of *MC1R* variants [79]. Blond and brown hair predictions are also relatively reliable (75–85%) but may show variation across different ethnic backgrounds. Black hair, being the most common worldwide, is typically predicted with high sensitivity but may have slightly lower specificity due to overlapping SNP effects [80].

Prediction models have been validated in European populations, where hair color diversity is greatest. However, performance in non-European populations can differ due to allele frequency differences and additional contributing variants. Ongoing research continues to expand databases and improve prediction accuracy across ancestrally diverse groups [81].

The utility of hair color prediction from biological traces hinges on the ability to generate accurate genotypes from compromised samples [82].

In cases involving severely degraded DNA, MPS-based typing offers advantages by enabling parallel analysis of multiple short DNA fragments and improving coverage at key SNP sites [67,83,84]. Still, probabilistic interpretation remains essential, particularly when prediction confidence falls below threshold values [82].

Although hair color is a relatively non-sensitive trait in forensic terms, the use of predictive models still raises questions about genetic privacy, the potential for profiling, and public trust. Moreover, probabilistic results should be presented with appropriate caveats, particularly when based on partial or degraded DNA samples [83].

### 3.3. Skin Pigmentation Prediction in Forensic Science from Biological Traces

The HIrisPlex-S model further incorporates skin color prediction and uses a total of 41 SNPs (Table 3), enabling a broader EVC profile from a single DNA sample. This comprehensive approach improves the informative value of FDP in real-world investigations [84].

While the SNP tables provide a comprehensive overview of markers used in FDP, performance varies significantly across panels and traits, especially under degraded-DNA conditions. The HIrisPlex-S system, which integrates eye, hair, and skin color prediction, has demonstrated robust performance in forensic contexts, including aged bone samples, ref. [59] with successful recovery of pigmentation profiles in over 55% of cases [9]. However, certain SNPs associated with skin tone (e.g., rs1426654 in *SLC24A5* and rs12913832 in *HERC2*) show reduced amplification success in highly degraded samples, leading to underperformance in intermediate pigmentation categories. VISAGE panels, designed for massively parallel sequencing, offer improved sensitivity and multiplexing, enabling better SNP recovery from LT-DNA and degraded skeletal remains. Inter-laboratory validation studies [59] confirm that VISAGE achieves higher reproducibility and lower dropout rates compared to HIrisPlex-S, particularly for skin color and ancestry inference. Nonetheless, both panels exhibit population-specific limitations: predictive accuracy is highest in European cohorts and declines in admixed populations, underscoring the need for inclusive reference datasets and ongoing calibration.

Skin pigmentation prediction from biological traces is an emerging and powerful facet of FDP, allowing forensic scientists to infer EVCs of unknown individuals when conventional identification techniques are unfeasible. Alongside eye and hair color, skin pigmentation provides essential descriptive features that can generate investigative leads from DNA alone. This is particularly relevant in cases involving unknown perpetrators, unidentified human remains, or degraded biological evidence [85].

Biological traces recovered from crime scenes can contain sufficient nuclear DNA to permit genotyping, and in such cases, the typing of SNPs associated with pigmentation traits [44]. Human skin color is primarily determined by the quantity and type of melanin produced by melanocytes in the basal layer of the epidermis. The density, distribution, and synthesis of melanin granules, regulated by a network of pigmentation genes, are key factors in determining skin tone [86]. These processes are influenced by several genes that affect melanosome formation, melanocyte biology, and melanin biosynthesis pathways [87]. Numerous genes have been associated with variations in skin pigmentation, many of which have been included in forensic prediction panels. Furthermore, skin color is a continuous trait influenced by both genetic and environmental factors (i.e., sun exposure, tanning behavior, and age can affect observed skin tone) [88]. Its prediction from DNA is complex due to its polygenic nature, wide variation across populations, and the influence of evolutionary history. Despite this, robust predictive models have been developed, especially those designed to distinguish between broad pigmentation categories (light, intermediate, dark) across major global ancestries [89]. This classification system simplifies the continuous nature of skin tone into practical categories for forensic investigation. The model utilizes logistic regression trained on a large reference dataset that includes individuals from diverse ancestral backgrounds [90].

In validation studies, HIrisPlex-S demonstrated high accuracy, particularly in predicting light and dark pigmentation. Intermediate skin tones are more challenging due to greater phenotypic and genetic diversity and less clear boundaries between categories. The prediction accuracy is also influenced by the genetic background of the individual: for example, predictions are more reliable in populations of European or sub-Saharan African descent, and less so in admixed or South Asian populations [81].

The prediction of skin color from LT-DNA is a promising yet complex frontier in forensic genetics. LT-DNA samples are inherently prone to degradation and contamination [70]. These factors can compromise the integrity of genetic markers, particularly SNPs used in pigmentation prediction models such as HIrisPlex-S. Indeed, LT-DNA samples frequently result in incomplete genetic profiles, which limits the number of informative SNPs available for analysis. This can hinder the statistical confidence of phenotype predictions, especially in individuals with intermediate or admixed ancestry, where subtle genetic variations play a significant role in pigmentation. Advanced probabilistic models and imputation techniques are being developed to address these limitations, but their effectiveness remains contingent on the quality and completeness of the input DNA [9].

Despite these challenges, LT-DNA-based skin color prediction remains a valuable tool in forensic investigations. When used responsibly, it can provide critical leads in otherwise unsolvable cases, offering a glimpse into the physical appearance of unknown individuals and narrowing suspect pools in a scientifically grounded manner.

### 3.4. Freckles and Sun Sensitivity Prediction in Forensic Science from Biological Traces

Among the suite of the prediction of EVCs from biological traces, freckles and sun sensitivity may seem minor at first glance, but they can contribute meaningfully to the construction of a biogeographic and phenotypic profile of an unknown individual [91]. Freckles (ephelides) are small, pigmented macules that typically appear on sun-exposed areas of fair skin. Sun sensitivity refers to a person’s susceptibility to burning rather than tanning upon UV exposure, typically resulting from lower eumelanin levels and higher pheomelanin content [86]. These traits, rooted in pigmentation biology, are closely associated with genes involved in melanin synthesis and regulation, particularly the *MC1R* gene, located on chromosome 16 [92]. Several common *MC1R* variants (e.g., R151C, R160W, D294H) are collectively referred to as “R alleles” and are associated with the red hair color (RHC) phenotype, but also contribute independently to freckling and UV sensitivity, even in individuals without red hair [93]. Other genes, such as *ASIP*, *TYR*, and *IRF4*, may modulate freckling and pigmentation patterns, although their effects are smaller and often population-specific [94].

With appropriate genetic analysis, forensic scientists can derive probabilistic predictions about whether an individual is likely to have freckled skin and/or increased sensitivity to sunlight. The relative SNPs are included in comprehensive forensic phenotyping tools such as HIrisPlex-S [85]. These models use multinomial logistic regression or ML classifiers trained on large, multi-ethnic datasets [51,95]. For freckles, individuals are categorized as likely or unlikely to have them based on the presence of specific MC1R variants and other associated SNPs [3,96,97,98,99]. For sun sensitivity, prediction involves assessing genetic profiles associated with reduced melanin production and increased burn tendency (high likelihood of sunburn with minimal tanning, moderate sensitivity, low sensitivity/likely to tan) [100].

Prediction accuracy for freckles and sun sensitivity is generally good when the relevant SNPs are reliably genotyped, and the individual belongs to a well-represented population in the model’s training data (typically of European ancestry) [51]. For instance, freckle prediction can reach accuracies above 75–80%, particularly when individuals carry two or more non-functional MC1R alleles. Sun sensitivity prediction is somewhat more variable due to the continuous nature of the phenotype and its interaction with environmental factors (e.g., lifetime sun exposure, behavior, geography) [101].

However, there are several limitations: -polygenic complexity: many small-effect genes and gene-environment interactions contribute to these traits; -environmental influence: freckling can be influenced by UV exposure history, making it a dynamic, rather than strictly genetically determined, trait; -population bias: predictive models perform best in individuals of European ancestry, where pigmentation variation is highest and best studied; accuracy drops in admixed or underrepresented populations; -LT-DNA [100].

### 3.5. Facial Morphology Prediction in Forensic Science from Biological Traces

In the field of FDP, the prediction of facial morphology, the shape and structure of the human face, represents a frontier with considerable scientific promise and complex challenges [102]. While traits like eye, hair, and skin color have reached relatively high predictive reliability, facial morphology prediction is more intricate due to the polygenic and multifactorial nature of facial features. However, recent advances in genomics, 3D facial imaging, and ML models are progressively transforming facial prediction from a speculative vision into an emerging forensic tool. The ability to reconstruct aspects of a person’s facial structure from biological traces such as blood, saliva, or touch DNA could significantly support investigations, particularly when no biometric or documentary information is available [102,103].

Facial morphology is a highly heritable trait governed by the interaction of hundreds of genes and regulatory elements. GWAS have identified dozens of loci associated with specific facial traits, including: facial width and height, nasal bridge length and tip projection, lip thickness, chin prominence, brow ridge shape, mandibular and maxillary dimensions [6,104].

Key genes such as *PAX3*, *EDAR*, *DCHS2*, *RUNX2*, and *PRDM16* have been linked to facial development, often through their role in craniofacial growth, tissue differentiation, and skeletal patterning. Variants in these genes influence the position and projection of facial landmarks that define individual appearance. Notably, the expression of these traits is further modulated by age, sex, ancestry, and environmental influences (e.g., nutrition, trauma), which makes prediction from DNA alone inherently probabilistic [105,106].

In addition to genotyping, the ancestry and sex of the donor are determined, as both play pivotal roles in shaping facial morphology. Ancestry informs baseline structural patterns common in different populations (e.g., nasal breadth, cheekbone prominence), while sex influences sexually dimorphic traits such as jaw angle and brow thickness [107].

To transform genetic data into facial predictions, researchers apply ML techniques trained on large databases of paired genotype and 3D facial imaging data. These datasets often consist of thousands of individuals with detailed facial scans and genome-wide SNP data. Key modeling approaches include partial least squares regression (PLSR) to link SNPs to principal components of facial variation; convolutional neural networks (CNNs) to analyze facial geometry from genetic and demographic inputs; deep generative models (e.g., variational autoencoders or GANs) to create realistic 3D face renderings from DNA data [108].

Recent examples include the FaceBase project, VisiGen, and research from the Penn State Forensic Anthropology Lab, which has shown that genetic data can explain 10–20% of the variance in certain facial traits, modest but meaningful progress toward practical forensic use [109,110]. Despite promising advances, current models explain only a modest proportion of facial trait variance, typically between 10–20%, with substantial limitations in predicting features influenced by environmental and developmental factors. Traits such as facial asymmetry, age-related changes, and expression dynamics remain poorly captured by genotype-based models. This modest predictive power underscores that current models are research-grade and unsuitable for operational forensic deployment.

The predictive power of facial trait modeling is strongest when combined with known ancestry, sex, and age estimates, particularly in young adults, whose facial structures are relatively stable. Nonetheless, these models often lack precision at the individual level and are more effective at generating composite depictions or narrowing demographic search pools than at identifying exact facial characteristics. Reproducibility remains a major challenge in facial morphology prediction. Variability in imaging techniques, SNP panels, and population structure can lead to inconsistent results across studies. Moreover, the lack of standardized validation protocols and limited availability of forensic-grade datasets restrict the generalizability of current models. According to recent publications, the highest predictability is observed for traits such as nose width, facial width, intercanthal distance (the distance between the eyes), and lip thickness. Moderate predictability applies to features like brow ridge prominence, chin shape, and nasal tip projection. In contrast, traits such as facial asymmetry, expression-related features, and age-related morphological changes exhibit low predictability [46,111].

In forensic contexts, these predictions are used as investigative leads rather than as confirmatory evidence. Reproducibility remains limited due to variability in imaging protocols, SNP panels, and population structure. Most data sets are European-biased, reducing generalizability and increasing misclassification risk in admixed populations. Future efforts should focus on expanding training datasets to include diverse populations, integrating multi-omics data to capture non-genetic influences, and establishing standardized pipelines for model development and validation to enhance reproducibility and forensic reliability. For example, in cold cases, a predicted facial image may be released to the public to generate tips. In mass disaster scenarios, facial predictions can assist in identifying unknown victims when traditional methods, such as fingerprinting or dental records, are unavailable. Similarly, in cases involving unidentified remains, predicted facial features can complement skeletal reconstructions and ancestry estimations, enhancing the overall identification process.

Facial prediction from LT-DNA represents a cutting-edge intersection of forensic genetics and computational modeling. Facial predictions derived from LT-DNA are probabilistic rather than definitive and are best used to generate investigative leads rather than serve as conclusive evidence [3,102]. Indeed, despite promising advances, current models explain only 10–20% of variance in facial traits, which is insufficient for individual-level reconstruction. These outputs remain research-grade and are not forensically deployable. Reproducibility is constrained by differences in imaging protocols, SNP panels, and population structure. Beyond technical limitations, predicting facial morphology raises significant ethical concerns. Unlike eye, hair, and skin color, facial features are closely linked to ancestry and identity, making them particularly sensitive. Generated facial composites risk being misinterpreted as accurate likenesses, potentially leading to investigational bias or discrimination. These challenges underscore the need for transparent communication of uncertainty, strict governance, and clear disclaimers when using such predictions as investigative leads rather than confirmatory evidence.

### 3.6. Age Prediction in Forensic Science Based on Biological Traces

Estimating chronological age from biological traces has become a valuable forensic tool, particularly when conventional identifiers are unavailable [112]. Unlike skeletal assessments, molecular approaches rely on biomarkers that change predictably over time [113]. Among these, DNA methylation at specific CpG sites is the most reliable indicator of age, outperforming earlier methods based on telomere length or mtDNA mutations, which showed high variability and limited forensic utility [114,115,116].

Epigenetic clocks exploit methylation changes at selected loci to predict age with high accuracy. Forensic models prioritize simplicity and robustness for degraded or LT-DNA. A widely used approach by Bekaert et al. employs four CpG sites in genes such as *ELOVL2*, *FHL2*, *KLF14*, and *TRIM59*, achieving mean absolute deviations of about 3–4 years in blood samples. *ELOVL2* is particularly informative due to its consistent methylation increase across tissues, including blood, saliva, and buccal cells [117]. The VISAGE Consortium has further advanced this field by creating multiplex assays compatible with NGS, enabling sensitive and robust age estimation from trace DNA. *ELOVL2*, in particular, has emerged as a key biomarker due to its consistent methylation increase with age across multiple tissue types, including blood, saliva, and buccal cells [118].

One of the strengths of DNA methylation-based age prediction is its applicability across various forensic sample types [119]. Blood and saliva are most commonly used due to their higher DNA yield and stable methylation profiles. Semen requires tissue-specific models, as methylation patterns vary significantly between tissues. Touch DNA, derived from epithelial cells, presents greater challenges due to low DNA quantities, but ongoing research is improving its feasibility. Even skeletal remains can be analyzed for methylation-based age estimation, offering valuable insights in cold cases or mass grave investigations [112,120].

In forensic casework, age prediction serves multiple roles. It can assist in suspect profiling when no known individual is linked to the biological evidence, providing an estimated age range to guide investigations. In victim identification, especially in cases involving unidentified remains, age estimates help correlate findings with missing persons databases. Additionally, age prediction is sometimes used in legal and immigration contexts to determine whether individuals are minors, although this application remains ethically and legally contentious [121,122].

Technological platforms for methylation analysis include pyrosequencing, quantitative PCR, and NGS. Pyrosequencing remains popular for targeted assays because of its cost-effectiveness and compatibility with forensic workflows, while NGS offers higher sensitivity and multiplexing for degraded samples. Recent VISAGE Consortium developments integrate methylation-based age estimation into multiplex panels, enabling simultaneous prediction of age and other traits from trace DNA [123,124].

Despite these advances, LT-DNA poses challenges such as allelic dropout and incomplete methylation profiles, which can reduce accuracy [125]. Optimized assays using short amplicons, replicate testing, and probabilistic models help mitigate these issues [126]. When combined with strict quality control and robust statistical frameworks, methylation-based age prediction provides actionable investigative leads, even from compromised samples [127].

### 3.7. Ancestry Prediction in Forensic Science from Biological Traces

Ancestry prediction from biological traces has become a powerful tool in forensic science, offering critical insights into the biogeographical origins of unknown individuals when traditional identification methods are unavailable [128]. This approach is particularly valuable in criminal investigations, mass disasters, and the identification of unidentified remains, where DNA evidence may be the only clue. Unlike cultural or self-reported ethnicity, forensic ancestry inference relies on genetic markers that reflect population-level differences shaped by evolutionary history, migration, and genetic drift. These markers, primarily SNPs, are distributed across the genome and exhibit frequency patterns that vary among continental and subcontinental populations [129].

The foundation of ancestry prediction lies in the analysis of ancestry-informative markers (AIMs), SNPs selected for their high allele frequency differences between populations. Panels such as the SNPforID 34-plex or others, such as the Precision ID Ancestry Panel, include hundreds of AIMs optimized for forensic use. These panels can distinguish major continental ancestries (e.g., African, European, East Asian, Native American, South Asian) and, in some cases, provide finer resolution within regions [130,131]. The VISAGE Consortium has developed advanced multiplex assays compatible with MPS, enabling ancestry inference from degraded or LT-DNA samples commonly encountered in forensic contexts [59,132].

Despite the promise of ancestry prediction, several challenges must be addressed, particularly when working with LT-DNA. However, recent advances in sequencing sensitivity and bioinformatic tools have improved the robustness of ancestry inference from trace samples. Probabilistic models and ML algorithms can now integrate partial genotypes and assign ancestry with high confidence, even from compromised DNA [90,133].

In forensic casework, ancestry prediction serves multiple purposes. It can help narrow suspect pools by providing investigators with information about the likely population background of an unknown individual. In missing persons cases, ancestry estimates can guide comparisons with databases and assist in facial reconstruction efforts. In mass disaster scenarios, ancestry inference can support victim identification when other biological or contextual information is lacking. Importantly, ancestry prediction is not intended to identify individuals directly but to provide investigative leads that complement other forensic evidence [134].

The accuracy of ancestry prediction depends on several factors, including the number and informativeness of SNPs used, the diversity of reference populations, and the complexity of individual genetic backgrounds. Admixed individuals, those with ancestry from multiple populations, pose particular challenges, as their genetic profiles may not align neatly with reference categories. To address this, modern forensic tools incorporate admixture analysis and ancestry deconvolution, estimating proportional ancestry contributions from different populations. These methods enhance the interpretability of results and reduce the risk of misclassification [135].

As forensic genomics continues to evolve, ancestry prediction from biological traces, especially LT-DNA, will play an increasingly important role in investigative workflows. With ongoing improvements in marker panels, sequencing technologies, and computational methods, the ability to infer ancestry from even the most challenging samples is becoming more accurate and accessible, offering valuable insights in the pursuit of justice.

## 4. Integrating ML with LT-DNA in FDP

The integration of ML into FDP represents a transformative advancement in the ability to predict EVCs from genetic material, particularly in challenging cases involving LT-DNA. LT-DNA samples, often recovered from trace biological material such as touch DNA or degraded remains, contain minimal quantities of DNA and are prone to allelic dropout, contamination, and stochastic effects. These limitations pose significant challenges for traditional statistical models, which often rely on complete and high-quality genotypic data. ML, with its capacity to handle high-dimensional, noisy, and incomplete datasets, offers a powerful solution for enhancing phenotype prediction from LT-DNA [95,136]. It is important to clarify that we define ML as a class of algorithms capable of learning complex, often non-linear relationships from data without relying on pre-specified parametric forms. Examples include Random Forests, Support Vector Machines, Gradient Boosting, and deep neural networks. In contrast, traditional statistical models, such as logistic regression, assume a fixed functional form and are widely used in operational FDP systems (e.g., IrisPlex, HIrisPlex-S, VISAGE). While logistic regression is sometimes grouped under supervised learning, it is not considered ML in the contemporary sense of high-dimensional, non-linear modeling.

### 4.1. Common ML Algorithms and Their Forensic Applications

ML models are particularly well-suited to forensic genomics due to several key factors: the high dimensionality of genomic data, the complex and non-linear relationships between SNPs and phenotypic traits, and the need to integrate diverse data types, including genotype, gene expression, and epigenetic modifications such as DNA methylation. Commonly used ML algorithms in FDP include Random Forests (RFs), which are valued for their robustness and interpretability; Support Vector Machines (SVMs), which perform well with small sample sizes and high-dimensional data; Gradient Boosting Machines (GBMs), which often outperform RFs in accuracy; and deep learning models such as neural networks (NNs), which are capable of modeling intricate genotype–phenotype relationships. Simple models like k-nearest neighbors (KNN) can also be effective in small datasets, particularly when combined with feature selection techniques [137,138]. Recent comparative studies provide quantitative evidence of ML performance in FDP. Katsara et al. [95] evaluated RFs, SVMs, and GBMs for eye color prediction using HIrisPlex SNPs in 1200 individuals. RF achieved an AUC of 0.93 for blue/brown classification, outperforming SVMs (AUC = 0.91) and logistic regression (AUC = 0.89) under 10-fold cross-validation. For skin color prediction, Zaorska et al. [91] applied neural networks to a Polish cohort (n = 800), reporting precision = 0.82 and recall = 0.79 for light pigmentation, compared to RF precision = 0.78 and recall = 0.75. VISAGE validation studies further demonstrated that ML-based genotype imputation improved SNP recovery from LT-DNA samples by 12–15%, enabling phenotype prediction from partial profiles. These findings underscore the advantage of ML in handling incomplete and noisy data, particularly in LT-DNA contexts (Table 4).

While deep learning models achieved slightly higher accuracy for complex traits, their lack of interpretability raises concerns for forensic admissibility, making RF and SVM preferred in operational contexts.

A critical challenge in applying ML to FDP is data imbalance and overfitting. Most training datasets, such as HIrisPlex and VISAGE, are predominantly composed of individuals of European ancestry, which can bias predictions and reduce accuracy in admixed or underrepresented populations [95,139]. Overfitting occurs when models capture noise or population-specific patterns rather than generalizable features, leading to poor performance on forensic casework samples.

To mitigate imbalance and overfitting, strategies such as feature selection (e.g., LASSO, recursive feature elimination) and regularization techniques (ridge regression, dropout layers in neural networks) are employed. These approaches improve generalization and reduce variance when working with high-dimensional genomic data.

These approaches collectively enhance robustness when working with LT-DNA, where incomplete and noisy profiles are common. However, further research is needed to validate these methods across diverse populations and forensic-grade datasets.

To provide a concise overview, Table 5 summarizes commonly used ML algorithms in FDP, their input data types, dataset sizes, predicted traits, and reported performance metrics. These results highlight that Random Forest and Gradient Boosting consistently achieve high AUC values for eye color prediction, while neural networks show promise for complex traits such as skin pigmentation. Deep learning approaches applied to facial morphology explain only a modest proportion of variance (10–20%), underscoring the need for larger and more diverse training datasets. For age estimation, regularized regression models using methylation data achieve mean absolute deviations of approximately 3–4 years, demonstrating their forensic utility.

### 4.2. Logistic Regression vs. Random Forest vs. Deep Learning in FDP: Accuracy, Data Require-Ments, and Admissibility

Operational FDP panels such as IrisPlex, HIrisPlex-S, and VISAGE rely on multinomial logistic regression because of its interpretability, stability under limited SNP sets, and extensive forensic validation. In contrast, RF, SVM, GBM, and deep learning architectures (CNNs, GANs) are increasingly explored in research for modeling complex, non-linear genotype–phenotype relationships and integrating diverse data types, but they remain largely experimental in operational pipelines.

Comparative evidence suggests that ML models can offer incremental accuracy gains under controlled conditions. For example, RF achieved AUC ≈ 0.93 for eye color prediction, outperforming SVM (≈0.91) and logistic regression (≈0.89) in cross-validation on HIrisPlex SNPs [95]. Neural networks reported precision ≈ 0.82 and recall ≈ 0.79 for skin color in a Polish cohort, slightly higher than RF (precision ≈ 0.78, recall ≈ 0.75) [91]. However, these improvements often diminish in LT-DNA casework due to allelic dropout and incomplete profiles. Facial morphology pre-diction remains particularly challenging, with deep learning explaining only 10–20% of variance in research cohorts [7,109,110,111], insufficient for individual-level reconstruction.

Modeling assumptions differ markedly. Logistic regression assumes a fixed parametric form and works well with modest sample sizes, making it straightforward to validate. RF, SVM, and GBM capture non-linear interactions and tolerate incomplete data but require larger, diverse datasets and careful regularization to avoid overfitting [95]. Deep learning demands thousands of samples and rich paired inputs (e.g., genotype and 3D facial scans), otherwise risking spurious, population-specific signals [7,109,110,111].

Overfitting and population bias remain critical concerns. FDP training datasets are often European-ancestry-heavy, which can skew predictions and reduce generalizability in admixed populations [95,135]. Cross-validation alone is insufficient; external and inter-laboratory validation on forensic-grade LT-DNA is essential [44,59]. Imputation of missing SNPs using ML can improve completeness but must include uncertainty estimates, and imputed calls should never be reported as hard genotypes [139,140,141].

Interpretability and admissibility are decisive for forensic use. Courts favor transparent methods with known error rates and peer-reviewed acceptance [14]. Logistic regression meets these standards; RF and GBM can be partially explained using feature importance and SHAP/LIME [136], but these tools are not yet standardized for legal contexts. Deep learning poses greater interpretability challenges, limiting its current admissibility.

Logistic regression remains the operational standard for FDP due to its interpretability and validation history. ML approaches are promising for research but remain experimental until broader validation, inclusive datasets, and courtroom-ready interpretability are achieved.

### 4.3. Future Directions and Operational Readiness

One of the most critical applications of ML in LT-DNA analysis is genotype imputation. Due to dropout events, many SNPs may be missing in LT-DNA samples. ML-based imputation tools such as Beagle, IMPUTE2, and deep learning-based networks can reconstruct missing genotypes with high accuracy, enabling more complete input data for phenotype prediction. Additionally, ML models are inherently more tolerant of noise and missing data than traditional approaches. Algorithms like RF and GBM can manage incomplete datasets by focusing on the most informative loci, while feature selection methods such as LASSO (Least Absolute Shrinkage and Selection Operator) and recursive feature elimination help reduce dimensionality and improve model performance [140].

Another promising strategy is the use of synthetic data augmentation to enhance model training. Techniques such as Synthetic Minority Oversampling Technique (SMOTE) or Generative Adversarial Networks (GANs) can simulate plausible low-quality or rare-case samples, thereby improving the generalizability of ML models to real-world forensic scenarios. This is particularly valuable in FDP, where the availability of high-quality, annotated training data is often limited [139,141].

The application of ML in FDP is already evident in tools like HIrisPlex and HIrisPlex-S, which use logistic regression and probabilistic models to predict eye, hair, and skin color from SNP panels. However, as FDP expands to include more complex traits such as facial morphology, freckles, sun sensitivity, and age estimation, ML becomes increasingly indispensable. For example, deep learning models trained on large datasets of paired genotype and 3D facial imaging data have shown promise in predicting facial structure, while epigenetic clocks based on DNA methylation patterns use regression models to estimate chronological age with high accuracy, even from degraded or LT-DNA samples [95,142].

To ensure forensic reliability, ML models must be trained and validated on diverse, well-annotated datasets such as the 1000 Genomes Project or the UK Biobank. Cross-validation and external validation using real forensic case data are essential to assess model robustness under practical constraints. Moreover, ethical considerations must guide the deployment of ML in forensic contexts, particularly regarding privacy, consent, and the communication of probabilistic predictions [143].

In summary, the integration of ML with LT-DNA analysis significantly enhances the predictive power and applicability of FDP. By enabling accurate phenotype inference from compromised samples, ML-driven FDP provides valuable investigative leads in cases where traditional methods fall short, marking a new era in forensic science.

## 5. Ethical Frameworks, Limitations, and Future Directions in FDP from LT-DNA

**Figure 3:**
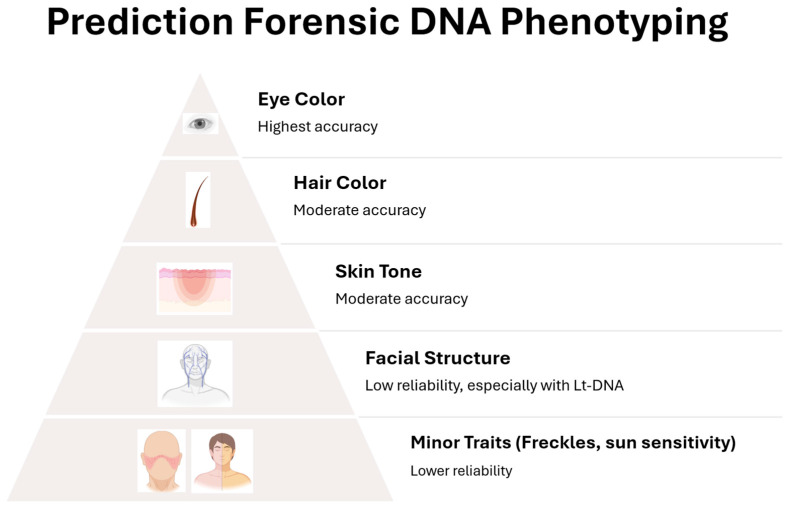
Relative prediction accuracy of EVCs from DNA, ranked by trait complexity. Eye color shows the highest accuracy (AUC > 0.9), followed by hair and skin color. Predictions for facial morphology, freckles, and sun sensitivity are less reliable, especially from LT-DNA. (Created with BioRender: www.biorender.com).

FDP has emerged as a powerful tool for predicting EVCs such as eye, hair, and skin color, as well as ancestry and age, from biological traces (Figure 3). However, when applied to LT-DNA samples, those containing minimal and often degraded genetic material, FDP faces significant technical, ethical, and interpretive challenges [144]. LT-DNA is particularly vulnerable to allelic dropout, contamination, and stochastic amplification errors, which can compromise the accuracy and reliability of phenotype predictions. These limitations are especially pronounced in complex traits like facial morphology, where prediction models rely on the integration of hundreds of genetic markers and are sensitive to even minor genotyping errors [1,145].

Prediction accuracy varies widely depending on the trait and the quality of the DNA. Eye color prediction remains the most robust, with AUC values exceeding 0.9 in European populations. Hair color and skin tone predictions are moderately accurate, while traits such as facial structure, freckles, and sun sensitivity are more difficult to predict reliably, particularly from LT-DNA. Moreover, most predictive models have been trained on individuals of European ancestry, limiting their applicability and accuracy in admixed or underrepresented populations. This population bias can lead to skewed or misleading results when applied in diverse forensic contexts [135] (Table 6).

Ethical and legal concerns further complicate the use of FDP in LT-DNA scenarios. Privacy remains a central issue, particularly under frameworks such as the EU General Data Protection Regulation (GDPR), which classifies genetic data as a special category requiring strict compliance with principles of lawfulness, purpose limitation, and data minimization. FDP applications in surveillance or public appeals raise heightened risks because they infer sensitive personal traits—such as ancestry or appearance—from biological material without consent. The secondary use of publicly available genomic data or biobank resources for forensic purposes introduces additional ethical challenges, including unclear consent scope, governance gaps, and potential erosion of public trust. Recent European debates on the European Health Data Space and U.S. discussions around investigative genetic genealogy highlight the tension between investigative utility and individual rights [69,146,147,148].

Algorithmic bias is another critical concern: ML models trained predominantly on European datasets often underperform for admixed or underrepresented populations, leading to inaccurate or misleading predictions. This bias can amplify inequities and undermine the legitimacy of FDP in diverse forensic contexts. To mitigate these risks, inclusive reference datasets, transparent validation across populations, and clear reporting of limitations are essential [149,150].

Court admissibility standards further demand rigorous attention. Under frameworks such as Daubert and Rule 702 in the U.S., and proportionality principles in Europe, FDP evidence must demonstrate scientific validity, known error rates, and peer-reviewed acceptance. Uncertainty should be communicated explicitly through probabilistic outputs, confidence intervals, and verbal scales, avoiding deterministic language that could mislead investigators or courts. Failure to convey uncertainty risks investigational bias or wrongful suspicion, particularly in high-stakes cases [14,151].

Finally, public perception and informed consent remain pivotal. Surveys and stakeholder interviews in Europe and the U.S. reveal mixed attitudes toward FDP, with concerns about genetic privacy, discrimination, and misuse. Transparent communication of capabilities and limitations, coupled with robust governance and consent frameworks, is essential to maintain trust and ensure that FDP serves justice without compromising fundamental rights [152,153].

To address these concerns, ethical frameworks must evolve alongside technological advancements. Clear guidelines are needed to govern the use of FDP, particularly in cases involving LT-DNA, where the margin for error is higher. Transparency in reporting, including the communication of prediction probabilities and confidence intervals, is essential to prevent misuse or misinterpretation. Additionally, the development of inclusive models trained in diverse populations is critical to ensuring fairness and accuracy across global forensic applications [154].

**Figure 4:**
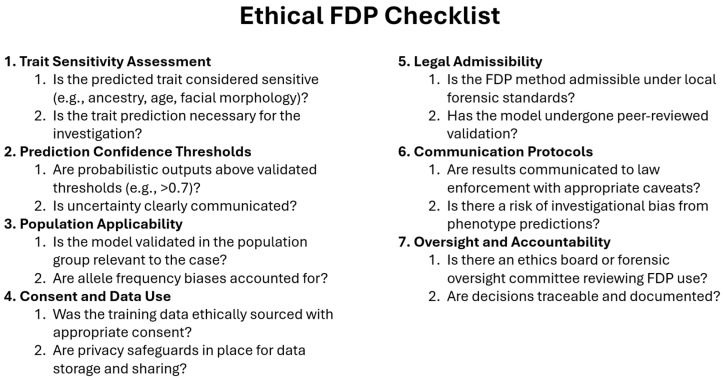
Ethical deployment checklist for FDP. Designed to assist forensic practitioners, legal professionals, and oversight bodies in evaluating scientific validity, privacy safeguards, and procedural accountability of FDP-based predictions.

To ensure that forensic FDP is applied responsibly and transparently, especially in cases involving LT-DNA, it is essential to establish operational safeguards that address both scientific limitations and ethical risks. To operationalize ethical safeguards, we propose the following checklist outlines key considerations for the ethical deployment of FDP in forensic investigations (Figure 4).

Looking ahead, several promising directions are emerging to enhance FDP from LT-DNA. One key area is the integration of multi-omics data, combining genomics with transcriptomics, epigenomics, and proteomics, to enable more dynamic and context-sensitive predictions. For example, integrating DNA methylation profiles can improve age estimation, while gene expression data may offer insights into lifestyle or environmental exposures. Another advantage is the development of portable and rapid phenotyping platforms, such as those based on Oxford Nanopore sequencing and edge-based ML inference, which could allow on-site analysis of LT-DNA in real time [155,156].

ML and artificial intelligence AI are also playing an increasingly significant role in FDP. ML models can manage noisy, incomplete data and are well-suited to the imputation of missing genotypes, a common issue in LT-DNA samples. Techniques such as SHAP values and LIME are being explored to improve model interpretability, which is crucial for courtroom credibility. Furthermore, synthetic data generation using generative adversarial networks (GANs) may help augment training datasets, improving model robustness in low-quality scenarios [157,158,159].

Ultimately, the future of FDP in LT-DNA contexts depends on balancing scientific innovation with ethical responsibility. By addressing current limitations, enhancing model inclusivity, and ensuring transparent communication, forensic science can harness the full potential of DNA phenotyping while safeguarding individual rights and public trust.

## 6. Conclusions

The intersection of LT-DNA analysis and ML is poised to revolutionize FDP. While ML approaches show promise for improving phenotype prediction from LT-DNA, most operational systems currently rely on logistic regression, which remains the forensic standard due to its interpretability and validation status. By enabling the prediction of EVCs from minimal and often degraded biological material, this integration expands the investigative potential of forensic science beyond traditional identity matching. ML models, with their ability to manage high-dimensional, noisy, and incomplete data, are particularly well-suited to the challenges posed by LT-DNA. They facilitate genotype imputation, enhance trait prediction accuracy, and support probabilistic interpretation of complex phenotypes such as facial morphology and age.

Despite these advances, significant limitations remain. Prediction accuracy varies by trait and is often reduced in LT-DNA due to allelic dropout and partial genotyping. Moreover, the majority of current models are trained on European-ancestry datasets, limiting their generalizability across diverse populations. Ethical concerns, including privacy, consent, and the risk of overinterpreting probabilistic predictions, must be addressed through robust legal and regulatory frameworks.

Looking ahead, the future of FDP in LT-DNA contexts lies in the integration of multi-omics data, the development of portable and rapid sequencing technologies, and the refinement of interpretable ML models. These innovations, coupled with efforts to diversify training datasets and standardize forensic protocols, will be essential for transitioning FDP from experimental research to routine forensic practice.

To strengthen future research and operational readiness, we recommend the following actions:

- Validation across diverse populations: Systematically validate ML-FDP models on non-European and admixed cohorts to mitigate algorithmic bias and improve global applicability.
- Integration of multi-omics data: Incorporate DNA methylation, transcriptomics, and proteomics into predictive frameworks to enhance accuracy for complex traits such as age and facial morphology.
- Portable sequencing workflows: Develop rapid, field-deployable FDP solutions using portable sequencing platforms (e.g., Oxford Nanopore) combined with edge-based ML inference for on-site analysis.
- Interpretability and transparency: Establish forensic ML interpretability standards using explainable AI tools (e.g., SHAP, LIME) to ensure transparency, accountability, and courtroom admissibility.
- Global collaboration: Promote international initiatives to create inclusive reference datasets and harmonized validation protocols across laboratories, ensuring reproducibility and fairness.

These steps will accelerate the transition of FDP from experimental research to operational forensic practice while safeguarding ethical and legal principles.

## Figures and Tables

**Table 1:** This table summarizes six SNPs located on various chromosomes, each associated with genes involved in eye color prediction.

SNP ID	Gene	Chromosome	Position	Reference Genome
rs12913832	*HERC2*	15	28120472	GRCh38 38.1/141
rs1800407	*OCA2*	15	27985172	GRCh38 38.1/142
rs12896399	*LOC105370627*	14	92307319	GRCh38 38.1/141
rs16891982	*SLC45A2*	5	33951588	GRCh38 38.1/141
rs1393350	*TYR*	11	89277878	GRCh38 38.1/141
rs12203592	*IRF4*	6	396321	GRCh37 37.1/131

**Table 2:** This table summarizes twenty-four SNPs located on various chromosomes, each associated with genes involved in eye and hair color prediction.

SNP ID	Gene	Chromosome	Position	Reference Genome
rs312262906 (rs796296176)	*MC1R*	16	89919342	GRCh38.p7 38.3/149
rs11547464	*MC1R*	16	89919683	GRCh38 38.1/141
rs885479	*MC1R*	16	89919746	GRCh38 38.1/141
rs1805008	*MC1R*	16	89919736	GRCh38 38.1/141
rs1805005	*MC1R*	16	89919436	GRCh38 38.1/141
rs1805006	*MC1R*	16	89919510	GRCh38 38.1/141
rs1805007	*MC1R*	16	89919709	GRCh38 38.1/141
rs1805009	*TUBB3*	16	89920138	GRCh38 38.1/141
rs201326893	*MC1R*	16	89919714	GRCh38 38.1/141
rs2228479	*MC1R*	16	89919532	GRCh38 38.1/141
rs1110400	*MC1R*	16	89919722	GRCh38 38.1/141
rs28777	*SLC45A2*	5	33958854	GRCh38 38.1/141
rs16891982	*SLC45A2*	5	33951588	GRCh38 38.1/141
rs12821256	*KITLG*	12	88934558	GRCh38 38.1/141
rs4959270	*LOC105374875*	6	457748	GRCh37.p5 37.3/135
rs12203592	*IRF4*	6	396321	GRCh37 37.1/131
rs1042602	*TYR*	11	89178528	GRCh38 38.1/141
rs1800407	*OCA2*	15	27985172	GRCh38 38.1/142
rs2402130	*SLC24A4*	14	92334859	GRCh38 38.1/141
rs12913832	*HERC2*	15	28120472	GRCh38 38.1/141
rs2378249	*PIGU*	20	34630286	GRCh38.p7 38.3/151
rs12896399	*LOC105370627*	14	92307319	GRCh38 38.1/141
rs1393350	*TYR*	11	89277878	GRCh38 38.1/141
rs683	*TYRP1*	9	12709305	GRCh38 38.1/141

**Table 3:** This table summarizes forty-one SNPs located on various chromosomes, each associated with genes involved in eye and hair color prediction.

SNP ID	Gene	Chromosome	Position	Reference Genome
rs312262906 (rs796296176)	*MC1R*	16	89919342	GRCh38.p7 38.3/149
rs11547464	*MC1R*	16	89919683	GRCh38 38.1/141
rs885479	*MC1R*	16	89919746	GRCh38 38.1/141
rs1805008	*MC1R*	16	89919736	GRCh38 38.1/141
rs1805005	*MC1R*	16	89919436	GRCh38 38.1/141
rs1805006	*MC1R*	16	89919510	GRCh38 38.1/141
rs1805007	*MC1R*	16	89919709	GRCh38 38.1/141
rs1805009	*TUBB3*	16	89920138	GRCh38 38.1/141
rs201326893	*MC1R*	16	89919714	GRCh38 38.1/141
rs2228479	*MC1R*	16	89919532	GRCh38 38.1/141
rs1110400	*MC1R*	16	89919722	GRCh38 38.1/141
rs28777	*SLC45A2*	5	33958854	GRCh38 38.1/141
rs16891982	*SLC45A2*	5	33951588	GRCh38 38.1/141
rs12821256	*KITLG*	12	88934558	GRCh38 38.1/141
rs4959270	*LOC105374875*	6	457748	GRCh37.p5 37.3/135
rs12203592	*IRF4*	6	396321	GRCh37 37.1/131
rs1042602	*TYR*	11	89178528	GRCh38 38.1/141
rs1800407	*OCA2*	15	27985172	GRCh38 38.1/142
rs2402130	*SLC24A4*	14	92334859	GRCh38 38.1/141
rs12913832	*HERC2*	15	28120472	GRCh38 38.1/141
rs2378249	*PIGU*	20	34630286	GRCh38.p7 38.3/151
rs12896399	*LOC105370627*	14	92307319	GRCh38 38.1/141
rs1393350	*TYR*	11	89277878	GRCh38 38.1/141
rs683	*TYRP1*	9	12709305	GRCh38 38.1/141
rs3114908	*ANKRD11*	16	89317317	GRCh38.p14
rs1800414	*OCA2*	15	27951891	GRCh38 38.1/141
rs10756819	*BNC2*	9	16858086	GRCh38 38.1/141
rs2238289	*HERC2*	15	28208069	GRCh38 38.1/141
rs17128291	*SLC24A4*	14	92416482	GRCh38.p14
rs6497292	*HERC2*	15	28251049	GRCh38.p14
rs1129038	*HERC2*	15	28111713	GRCh38 38.1/141
rs1667394	*HERC2*	15	28285036	GRCh38 38.1/141
rs1126809	*TYR*	11	89284793	GRCh38 38.1/141
rs1470608	*OCA2*	15	28042975	GRCh38.p14
rs1426654	*SLC24A5*	15	48134287	GRCh38 38.1/141
rs6119471	*ASIP*	20	34197406	GRCh38.p14
rs1545397	*OCA2*	15	27942626	GRCh38.p14
rs6059655	*RALY*	20	34077942	GRCh38.p7 38.3/151
rs12441727	*OCA2*	15	28026629	GRCh38.p14
rs3212355	*MC1R*	16	89917970	GRCh38.p14
rs8051733	*DEF8*	16	89957798	GRCh38.p14

**Table 4:** Performance of ML algorithms for FDP traits across datasets.

Trait	Algorithm	Dataset	AUC	Precision	Recall	Reference
Eye color	Random Forest	HIrisPlex SNPs	0.93	0.90	0.88	[95]
Eye color	SVM	HIrisPlex SNPs	0.91	0.88	0.86	[95]
Skin color	Neural Network	Polish cohort	--- (*)	0.82	0.79	[91]
Skin color	Random Forest	Polish cohort	--- (*)	0.78	0.75	[91]

* AUC not reported for skin color in Zaorska et al. [91].

**Table 5:** Summary of ML algorithms applied in FDP: input type, dataset size, accuracy, and key references.

Algorithm	Input Type	Dataset Size	Trait(s) Predicted	Reported Accuracy (AUC/Precision/Recall)	Reference
Random Forest	SNP array (HIrisPlex)	~1200 individuals	Eye color (blue/brown)	AUC = 0.93; Precision = 0.90; Recall = 0.88	[95]
SVM	SNP array (HIrisPlex)	~1200 individuals	Eye color	AUC = 0.91; Precision = 0.88; Recall = 0.86	[95]
Neural Network	SNP array	~800 individuals	Skin color	Precision = 0.82; Recall = 0.79	[91]
Gradient Boosting	SNP array	~1200 individuals	Eye color	AUC = 0.92	[95]
Deep Learning (CNN)	SNP + 3D facial scans	~3000 individuals	Facial morphology	Explains 10–20% variance in facial traits	[7]
Elastic Net	DNA methylation (CpG sites)	~500 individuals	Age estimation	MAD ≈ 3.5 years	[117]

Accuracy metrics vary by population and validation method; most studies used cross-validation. IrisPlex and HIrisPlex-S use logistic regression, not ML in the contemporary sense.

**Table 6:** Comparative prediction accuracies for major traits across populations.

Trait	Category	Accuracy (%)	Notes
Eye color	Blue/Brown	>90	High reliability in Europeans
Eye color	Intermediate	70	Low sensitivity (1–2%)
Hair color	Red	85–90	Strong MC1R effect
Hair color	Blond/Brown	75–85	Moderate accuracy
Skin color	Light/Dark	80–85	Reliable in Europeans/African cohorts
Skin color	Intermediate	<70	Challenging, especially in admixed

## Data Availability

All data were included in the manuscript.

## Abbreviations

The following abbreviations are used in this manuscript:

FDP	Forensic DNA Phenotyping
LT-DNA	Low Template DNA
ML	Machine Learning
EVC	Externally Visible Characteristics
SNP	Single Nucleotide Polymorphism
STR	Short Tandem Repeat
GWAS	Genome-Wide Association Studies
NGS	Next-Generation Sequencing
MPS	Massively Parallel Sequencing
WGS	Whole-Genome Sequencing
PCR	Polymerase Chain Reaction
RFU	Relative Fluorescence Units
AT	Analytical Threshold
scDNA	Single-Cell DNA
PGS	Probabilistic Genotyping Software
AIMs	Ancestry-Informative Markers
CNN	Convolutional Neural Network
GAN	Generative Adversarial Network
SMOTE	Synthetic Minority Oversampling Technique
LASSO	Least Absolute Shrinkage and Selection Operator
PLSR	Partial Least Squares Regression
CpG	Cytosine-phosphate-Guanine (dinucleotide)
MAD	Mean Absolute Deviation
qPCR	Quantitative Polymerase Chain Reaction
VISAGE	VISible Attributes through GEnomics
AI	Artificial Intelligence
SHAP	SHapley Additive exPlanations
LIME	Local Interpretable Model-Agnostic Explanations
